# Case Report: Two-month-old infant with PHACE syndrome: facial hemangioma and severe complex coarctation of the aorta

**DOI:** 10.3389/fcvm.2025.1539168

**Published:** 2025-02-03

**Authors:** Sánchez-Ortiz David, Deiros-Bronte Lucía, De la Hoz-Marañón Lucía, Feito-Rodríguez Marta, Rey-Lois Juvenal, Salas-Mera Diana, Labrandero-De Lera Carlos, López-Gutiérrez Juan Carlos, Gutiérrez-Larraya Federico

**Affiliations:** ^1^ Cardiology Department, Hospital Universitario Puerta de Hierro Majadahonda, Madrid, Spain; ^2^ Molecular Mechanics of the Cardiovascular System Laboratory, Spanish National Center of Cardiovascular Research, Madrid, Spain; ^3^ Pediatric Cardiology Department, Hospital Infantil La Paz, Madrid, Spain; ^4^ Cardiology Department, Hospital General Universitario Doctor Balmis, Alicante, Spain; ^5^ Dermatology Department, Hospital Infantil La Paz, Madrid, Spain; ^6^ Cardiac Surgery Department, Hospital Infantil La Paz, Madrid, Spain; ^7^ General Surgery Department, Hospital Infantil La Paz, Madrid, Spain

**Keywords:** PHACE syndrome, hemangioma, aortic coarctation, aortic malformations, neural crest migration

## Abstract

**Background:**

PHACE syndrome is an uncommon disorder, marked by large segmental hemangiomas on the face and various developmental anomalies. Significant advancements have been made in its diagnosis, imaging, and understanding of complications since 1996.

**Case Summary:**

We describe the first diagnosis case of PHACE syndrome in a one-month and 19-day-old infant who presented with a large facial hemangioma and coarctation of the aorta originating from the left common carotid artery, along with an aberrant course of the right subclavian artery. The complementary diagnostic studies, their surgical correction, and their progression are described.

**Discussion:**

Given the rarity of the syndrome (the PHACE Syndrome International Clinical Registry and Genetic Repository has over 270 enrolled individuals) and the importance of early diagnosis of some anomalies it comprises, particularly cardiovascular anomalies, dissemination is considered crucial for general and pediatric cardiologists.

## Introduction

PHACE syndrome is a rare disorder characterized by large segmental facial hemangiomas and various developmental anomalies, such as posterior fossa malformations, large facial hemangiomas, anatomical abnormalities of the cerebral arteries, aortic coarctation and other cardiac anomalies, and ocular anomalies. Occasionally, sternal anomalies are also present, in which case the syndrome is referred to as PHACES. Recently, two additional manifestations have been added to the clinical spectrum of PHACE syndrome: stenosis of the vessels at the cranial base and longitudinal segmental dilations of the internal carotid artery. We report the first diagnosis in a two-month-old girl with a large facial hemangioma, aortic coarctation from the left common carotid artery, and an aberrant right subclavian artery. Diagnostic studies, surgical correction, and progression are detailed. Given the syndrome's rarity and the importance of early diagnosis, especially of cardiovascular anomalies, dissemination is crucial for general and pediatric cardiologists.

## Case description

The patient, a one-month and 19-day-old infant, term birth at 38 weeks and with non-consanguineous parents both 23 years old, was referred for dermatological evaluation from a secondary hospital due to a hemifacial hemangioma with progressive growth and extension over the past week, leading to a recommendation for transthoracic echocardiography to assess potential associated congenital cardiac disease. Upon physical examination, the patient clinically presented with a segmental V3 hemangioma, with lesions along the left mandibular branch, both labial commissures, tongue tip, and anterior cervical region, collectively extending over 7 cm. Decreased distal pulses were found in both lower limbs. The blood pressure recorded in the upper limbs was 95/68 mmHg, and the blood pressure in the lower limbs was 68/42 mmHg. She did not exhibit any cardiac symptoms. She had good weight gain, no hyperhidrosis, and no respiratory distress. No other findings were noted on physical examination.

Given the possibility of PHACE syndrome, an initial transthoracic echocardiogram was performed, revealing severe coarctation of the native aorta with a peak gradient of 70 mmHg and diastolic flow prolongation, aortic arch hypoplasia (proximal, distal transverse aortic arch and aortic isthmus 5.7 mm, 3 mm, 2.7 mm; with Z scores −2.9, −3.1 and −3 respectively), and ascending aorta dilation (13 mm, z score 3). The left ventricle was dilated with a borderline low ejection fraction (0.48). With these findings, the diagnosis was established, and the patient was admitted for comprehensive evaluation and treatment.

A transfontanellar ultrasound, an abdominal ultrasound, and an ophthalmological examination were performed, all yielding normal results. Additionally, a genetic analysis was requested through comparative genomic hybridization using a 60 K array to exclude chromosomal anomalies, and massive sequencing using a gene panel associated with vascular malformations was conducted.

**Figure 1:**
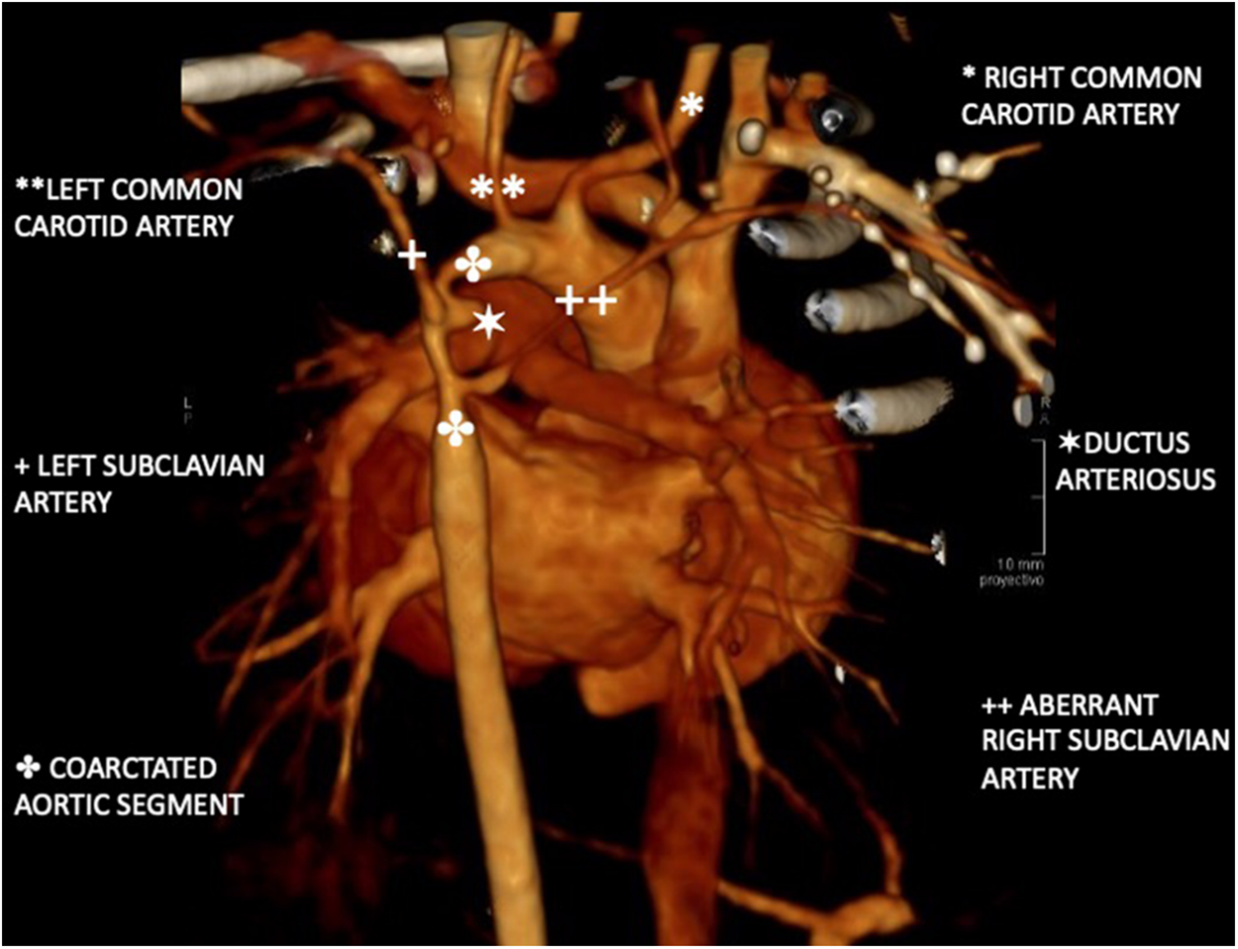
Three-dimensional reconstruction of aortic CT angiography. Periductal aortic stenosis extends 10 mm craniocaudally with a 1 mm diameter. Distal aorta measures 4 mm, widening to 5 mm at the diaphragmatic hiatus. An aberrant right subclavian artery collapses the esophagus. The left common carotid artery is reduced to 2 mm. at its origin. A partially filled ductus is observed with a diameter of 3 mm.

The vascular study was completed with a thoracic CT angiography (Figure 1), which showed severe periductal coarctation of the aorta and partial ductal patency. Periductal aortic stenosis was observed over an extension of approximately 10 mm craniocaudally with a diameter of 1 mm. Distally to the coarctation, the aorta presented a caliber of 4 mm, widening to 5 mm at the diaphragmatic hiatus. An aberrant right subclavian artery was identified, compressing the diaphragm as it traversed the mediastinum. The left common carotid artery was reduced in caliber, with an initial diameter of 2 mm (contralateral carotid diameter of 3.7 mm at its origin).

Multiple images compatible with hemangiomas were identified in a clinical context, located around the infraglottic trachea (which narrows at this level), between the origins of the left subclavian artery and vein, within the left parotid gland, the left facial region, posterior to the right thyroid lobe, and between the left carotid artery and internal jugular vein.

Following a multidisciplinary medical-surgical session discussion, it was decided to schedule aortic advancement surgery during the hospital stay. During the procedure, due to the length of the coarcted segment and abundant collateral circulation, aortic advancement was not feasible. Consequently, resection of the coarcted segment with end-to-end anastomosis and interposition of a Dacron patch was performed. The patient experienced a favorable outcome, allowing for early hospital discharge.

The lower limb pulses normalized following the procedure. The follow-up ultrasound revealed a residual gradient of 45 mmHg without diastolic flow prolongation. Consequently, it was decided to conduct close clinical and echocardiographic monitoring, with percutaneous dilation to be performed in the event of significant clinical or hemodynamic changes.

## Discussion

PHACE syndrome, an uncommon disorder with an unknown etiology, is characterized by large segmental hemangiomas on the face and a variety of developmental anomalies. The term “PHACE(S)” may be used when ventral defects like sternal clefts or supraumbilical raphe are present. Since its initial description in 1996 (1), there have been considerable advancements in diagnostic criteria, imaging guidelines, and understanding associated complications (2).

The exact incidence and prevalence of PHACE syndrome are not well-defined. The PHACE Syndrome International Clinical Registry and Genetic Repository have over 270 individuals enrolled.

The origin of PHACE syndrome is unidentified and appears to be a sporadic condition without familial occurrences. Some researchers suggest it may result from a somatic mosaic mutation in neural crest origin cells, crucial for embryogenesis and midline structural development. Midline defects in the sternum and brain, along with cervical and cerebral arterial anomalies, indicate that pathogenic events or developmental field defects likely occur between weeks 3 and 12 of gestation, coinciding with early vasculogenesis. PHACE might involve complex pathogenetic mechanisms, potentially related to gene-environment interactions, *in utero* hypoxia, or multifactorial origins (3).

The female predominance seen in PHACE cases led to hypotheses about an X-linked inheritance with possible male lethality. However, a study comparing 59 males with 213 females revealed no significant difference in phenotype severity, except for a slight increase in structural brain anomalies in males (4). Further, analyses of X-inactivation patterns in 31 females and their mothers showed no substantial skewing (5). Additionally, genome-wide copy number variation analysis within a cohort of 98 individuals disclosed no common deletions or duplications, complicating the understanding of the syndrome's origin further (6).

In PHACE syndrome, hemangiomas may initially appear as bruised or vasoconstricted patches at birth. These hemangiomas become more pronounced within the first few weeks of life and commonly exhibit a segmental distribution on the face. Infants with multiple facial segments affected have a higher risk of PHACE syndrome. Notably, large segmental hemangiomas on the scalp, trunk, or upper extremity may also indicate PHACE-associated congenital anomalies, even if no facial hemangioma is present (7).

While hemangiomas in PHACE syndrome are frequently proliferative, some patients may exhibit infantile hemangiomas with minimal or halted growth, characterized by a thin and telangiectatic appearance. These hemangiomas should be differentiated from nascent hemangiomas, which are often mistaken for port wine birthmarks before they enter the proliferative phase (2, 8).

A study examining 59 brain MRIs from individuals with PHACE syndrome found that 41% displayed structural brain anomalies. Unilateral cerebellar hypoplasia is the most common structural brain anomaly, typically on the same side as the arterial anomalies and hemangioma, and can sometimes be detected prenatally via routine fetal ultrasound. Recognizing the connection between cerebellar hypoplasia and PHACE facilitates earlier diagnosis and treatment (9). Other cerebellar anomalies associated with PHACE include Dandy-Walker variants and true Dandy-Walker malformation (8).

Regarding eye abnormalities, major diagnostic criteria also involve posterior segment anomalies, such as persistent fetal vasculature, “morning-glory” disc, peripapillary staphyloma, and optic nerve hypoplasia. Minor diagnostic criteria pertain to anterior segment anomalies, which may involve coloboma, iris hypoplasia, congenital cataracts, sclerocornea, and iris hypoplasia (2, 8).

Patients with PHACE syndrome face risks of congenital or acquired hypothyroidism. Congenital hypothyroidism, typically identified through newborn screening tests, may result from an absent or poorly developed pituitary gland (central hypothyroidism), congenital absence of the thyroid gland, or a lingual ectopic thyroid gland. An MRI can potentially identify congenital absence of the thyroid gland (2, 8).

In a study of 150 patients from the International PHACE Registry, 62 individuals (41%) exhibited cardiac anomalies, defined as intracardiac, aortic arch, or brachiocephalic vessel anomalies. Notably, 19% presented with coarctation of the aorta, 21% had an aberrant subclavian artery, 3% a vascular ring, and 13% a ventricular septal defect. Furthermore, 8% showed venous anomalies, and 6% had other structural heart defects. Conditions such as Tetralogy of Fallot, Holmes heart, and ectopia cordis have been reported in association with PHACE syndrome (10).

Coarctation in PHACE syndrome characteristically manifests as a long-segment transverse arch narrowing, often with adjacent aneurysmal dilation, contrasting with the common juxtaductal coarctation. Analysis of affected tissue reveals significant muscle cell and elastic fiber loss. Additional anomalies include right, double, and interrupted aortic arches. The prevalence of aberrant subclavian arteries necessitates careful evaluation, as traditional blood pressure assessments may be unreliable. Vascular rings may cause swallowing difficulties requiring surgical intervention (2, 8, 10). It is very important to consider the nature and extent of these anomalies, especially the exceptional extension of the coarcted segment and the involvement of subsidiary branches, as it impacts decision-making regarding surgical repair and follow-up.

The recognition and understanding of these anomalies and associations play a crucial role in the early diagnosis and management of PHACE syndrome, allowing healthcare providers to tailor assessments and interventions accordingly.

## Conclusions

**Figure 2:**
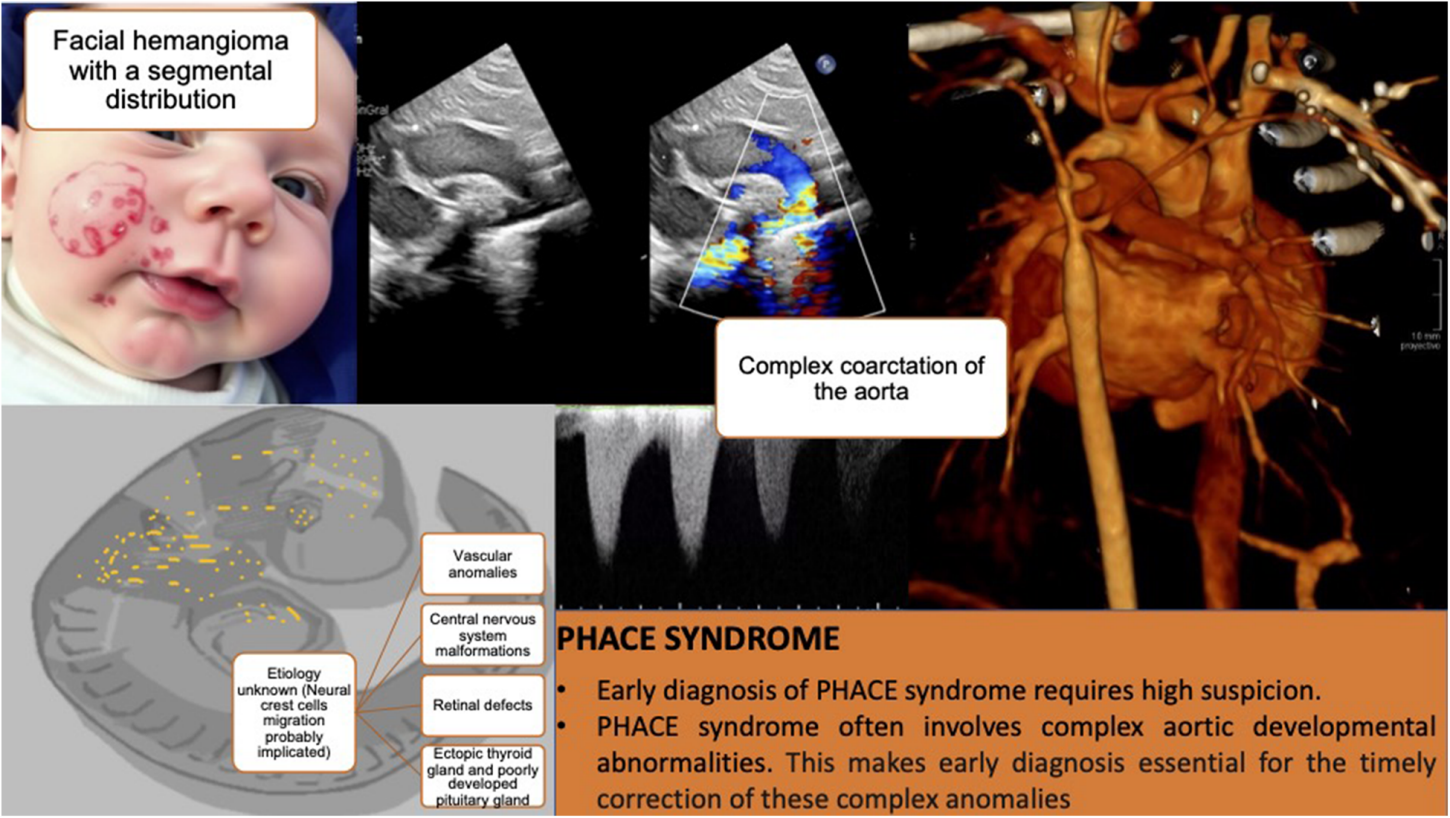
Composition that summarizes the main milestones of the case. The first panel shows an infant with a large facial hemangioma (the figure was created using an artificial intelligence tool and does not correspond to a real image). Subsequent panels present diagnostic images and key lessons.

The presented case highlights the importance of maintaining a high degree of suspicion for the early diagnosis of malformative syndromes such as PHACE syndrome, where the hemangioma serves as a red flag that, combined with a high degree of suspicion, allows for early therapeutic measures. Abnormalities of the brain, aorta, medium-sized arteries of the chest, neck and head are common and have the greatest potential to cause long-term morbidity. This is particularly relevant in the context of a complex aortic coarctation, which would have had more severe consequences if left untreated and is very difficult to diagnose prenatally due to fetal hemodynamics unless associated with identifiable anomalies in gestational screening ultrasounds, such as central nervous system anomalies (Figure 2).

## Data Availability

The original contributions presented in the study are included in the article/Supplementary Material, further inquiries can be directed to the corresponding author.
